# Decoupling Phase Separation and Fibrillization Preserves Activity of Biomolecular Condensates

**DOI:** 10.21203/rs.3.rs-6405673/v1

**Published:** 2025-04-29

**Authors:** Tharun Selvam Mahendran, Anurag Singh, Sukanya Srinivasan, Christian M. Jennings, Christian Neureuter, Bhargavi H. Gindra, Sapun H. Parekh, Priya R. Banerjee

**Affiliations:** 1 Department of Biological Sciences, The State University of New York at Buffalo, Buffalo, NY, 14260, USA; 2 Department of Physics, The State University of New York at Buffalo, Buffalo, NY, 14260, USA; 3 Department of Biomedical Engineering, University of Texas at Austin, Austin, TX, 78712, USA

## Abstract

Age-dependent transition of metastable, liquid-like protein condensates to amyloid fibrils is an emergent phenomenon of numerous neurodegeneration-linked protein systems. A key question is whether the thermodynamic forces underlying reversible phase separation and maturation to irreversible amyloids are distinct and separable. Here, we address this question using an engineered version of the microtubule-associated protein Tau, which forms biochemically active condensates. Liquid-like Tau condensates exhibit rapid aging to amyloid fibrils under quiescent, cofactor-free conditions. Tau condensate interface promotes fibril nucleation, impairing their activity to recruit tubulin and catalyze microtubule assembly. Remarkably, a small molecule metabolite, L-arginine, selectively impedes condensate-to-fibril transition without perturbing phase separation in a valence and chemistry-specific manner. By heightening the fibril nucleation barrier, L-arginine counteracts age-dependent decline in the biochemical activity of Tau condensates. These results provide a proof-of-principle demonstration that small molecule metabolites can enhance the metastability of protein condensates against a liquid-to-amyloid transition, thereby preserving condensate function.

## Introduction

Protein condensation via phase separation is facilitated by an interplay between chain solvation and multivalent sequence-encoded inter-molecular interactions^1, 2, 3, 4, 5, 6^. Typically, protein condensates possess physical properties akin to viscoelastic fluids^7, 8, 9^. In certain protein systems associated with neurodegenerative disorders (ND), including FUS, hnRNPA1, TDP-43, and Tau, phase separation is linked to age-dependent alterations in condensate dynamics, morphology, and structure—a process known as physical aging or maturation^10, 11, 12, 13, 14, 15, 16^. The aging of protein condensates is an emergent property of the system described in recent literature using the framework of a glass transition^8^, dynamical arrest of the dense phase^9^, and a fluid-to-amyloid fibril transition^17^. Importantly, several clinically relevant mutations in ND-linked proteins were found to accelerate the liquid-to-fibril transition of condensates^11, 12, 16, 18, 19^, indicating that protein phase separation may contribute significantly to disease-associated fibrillization^20, 21, 22, 23^. However, an open question remains: Are the thermodynamic driving forces governing protein phase separation and fibrillization related or distinct?

**Figure 1.**
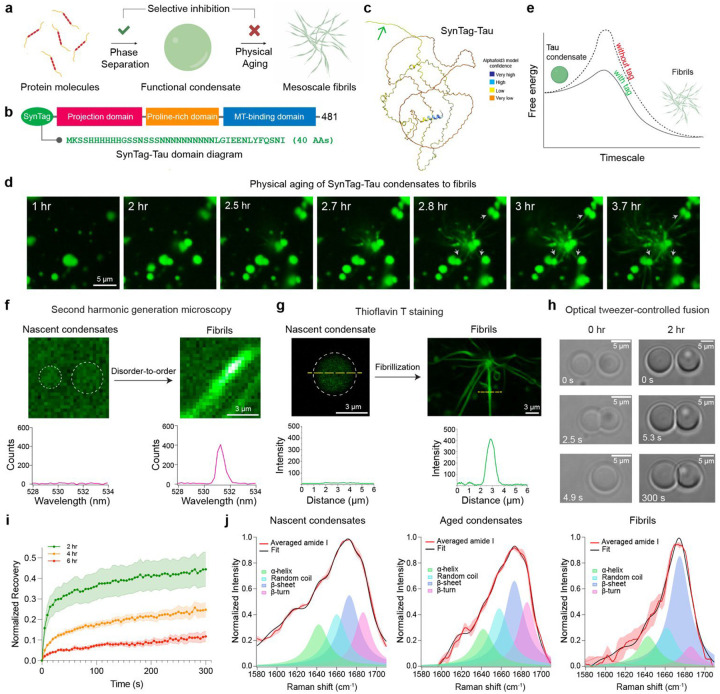
Engineered SynTag-Tau with a prionogenic tag undergoes phase separation coupled to fibril formation under cofactor-free quiescent conditions. **(a)** Schematic depicting the formation of phase-separated protein condensates and their time-dependent transition to form mesoscale amyloid fibrils. **(b)** Domain diagram of SynTag-Tau consisting of full-length Tau (2N4R isoform) with an N-terminal synthetic prionogenic tag (sequence provided in green). **(c)** Alphafold3^67^ predicted structure of SynTag-Tau. The green arrow marks the location of the tag. **(d)** Time-lapse imaging of Atto488-labeled SynTag-Tau condensates shows time-dependent morphological changes of the condensates to mesoscale fibrils. Grey arrows indicate the attachment of fibrils emerging from one condensate to neighboring condensates. Also, see **Supplementary Video 1**. **(e)** Schematic of a free energy diagram showing condensate-to-fibril transition with and without the prionogenic tag fused at the N-terminus of full-length Tau. **(f)** Second harmonic generation (SHG) microscopy shows the absence of molecular ordering in nascent SynTag-Tau condensates (indicated by dashed circles; sample age = 2 hours) and the presence of spatial orders in fibrils (sample age = 10 hours). **(g)** Thioflavin T (ThT) fluorescence line profiles from nascent SynTag-Tau condensates (indicated by a dashed circle; sample age = 2 hours) and fibrils (sample age = 10 hours). **(h)** Optical tweezer-controlled fusion of nascent SynTag-Tau condensates (sample age = 30 mins, indicated as the 0-hour time point) and condensates at 2 hours of age. **(i)** FRAP measurements of SynTag-Tau condensates at various time points as indicated. Intensity error bars were plotted based on the standard error of the mean (S.E.M.) at each time point and are represented as the shaded regions. **(j)** Peak fitting of averaged amide I spectra obtained from broadband coherent anti-Stokes Raman (BCARS) hyperspectral imaging shows the structural profile of SynTag-Tau in condensates at 2 hours of age (nascent condensates) and at 6 hours of age (aged condensates), as well as fibrils formed at 10 hours of age. For panel (d), the concentration of SynTag-Tau used is 24 μM with 10% PEG 8000. For panels (f), (g), and (j), the concentration of SynTag-Tau used is 12 μM with 10% PEG8000. For panels (h) and (i), the concentration of SynTag-Tau used is 12 μM with 7.5% PEG8000. The buffer composition in all samples here is 10 mM HEPES (pH 7.4), 50 mM NaCl, 0.1 mM EDTA, and 2 mM DTT. Wherever applicable, the concentration of Atto488-labeled SynTag-Tau is 250 nM, and the concentration of ThT is 50 μM. Each experiment was independently repeated at least three times.

Emerging experimental and computational results suggest that protein condensates are metastable relative to a globally stable solid state^9, 17, 24^. This may explain why stress granules hosting multiple aggregation-prone proteins become irreversible with prolonged persistence^11, 25^. It is plausible that active cellular processes and molecular chaperones, previously shown to regulate protein phase behavior^26, 27, 28, 29, 30, 31^, could counteract the age-dependent liquid-to-solid phase transitions of biomolecular condensates. Utilizing sequence engineering approaches for the prion-like low complexity domain of hnRNPA1, it was recently shown that the physical aging of protein condensates is sequence-encoded and partially distinct from the interactions that drive condensate formation^24^, leading to the postulate that distinct molecular grammars might exist for phase separation and physical aging. This indicates that macromolecules (such as proteins, RNAs, etc.) and/or small molecules that can selectively modulate sequence-specific interactions underlying amyloid formation could, in principle, buffer condensate liquid-to-solid transition independent of protein phase separation (Fig. 1a). Chemical targeting via small molecules has already been shown to modulate protein condensate formation and disassembly^26, 32, 33, 34^. With the growing interest in targeting aberrant biomolecular condensates with small molecules^35, 36, 37, 38, 39^, a key focus is on using small-molecule approaches to disentangle the molecular driving forces of phase separation from the physical aging of condensates associated with ND-linked proteins.

Motivated by these open questions, we sought to establish a model protein condensate system guided by the following criteria: (i) the ability to form fluid-like condensates that robustly age to globally stable solids under quiescent conditions, and (ii) condensates possess a tractable biochemical activity, enabling direct assessment of how physical aging affects condensate function. Establishing this benchmark will help differentiate between selectively disrupting condensate aging and broadly perturbing condensate formation, which could impact their biochemical activity. ND-linked proteins such as α-synuclein, FUS, and hnRNPA1 form condensates that undergo aging to fibrillar solids^12, 15, 17, 40^; however, the biochemical activities of condensates formed by these protein systems have not been established yet. Conversely, the microtubule-associated protein Tau, associated with a class of NDs called Tauopathies^41, 42, 43, 44, 45^, is known to form biochemically active condensates capable of microtubule (MT) assembly and stabilization implicated in intracellular trafficking in post-mitotic neurons^46, 47^. Importantly, multiple studies have hypothesized a link between the pathological cascade of Tau and Tau condensation, as pathogenic mutations have been shown to accelerate Tau condensate maturation^48, 49, 50, 51, 52^. These outstanding features provide a case for Tau as our model protein system. However, the aging of wild-type Tau condensates to amyloid fibrils is a kinetically sluggish process (**Supplementary Fig. 1**), hallmarked by the incredibly long timescale required for Tau aggregation and prion-like spread in vitro and in vivo^53, 54^. Tau is also well known for being highly soluble and recalcitrant to aggregation in vitro, often attributed to its hydrophilic nature^44, 55^. To enable Tau fibrillization within an experimentally accessible timescale, previous studies have used physicochemical perturbations including negatively charged cofactors like heparin, shear stress, and modifications to the native protein, such as truncations or phosphorylation^49, 51, 56, 57^. These strategies impose unintended effects on Tau condensation and their activity in MT assembly^46, 58^ independent of condensate aging.

Here, we use SynTag-Tau, which comprises the full-length Tau protein (2N4R isoform) and a 40-amino-acid prionogenic N-terminal tag. Serendipitously, we found that this synthetic prion-like low-complexity sequence substantially reduces the Tau condensate-to-fibril nucleation barrier. SynTag-Tau undergoes molecular crowding-dependent phase separation in vitro to form biochemically active liquid-like condensates, similar to the untagged wild-type Tau protein^49^. Importantly, unlike the untagged counterpart, SynTag-Tau condensates undergo physical aging to mesoscale amyloid fibrils in the timescale of hours under quiescent conditions and in the absence of polyanionic cofactors, shear stress, or perturbations to the core microtubule-binding domain. Using the SynTag-Tau system, we uncovered that conditions that promote protein phase separation commensurately accelerate condensate aging to amyloid fibrils. SynTag-Tau condensate interface nucleates fibril formation, yielding a disorder-to-order transition with gradual changes in the condensate’s dynamical and structural properties. These age-dependent transitions in SynTag-Tau condensate physical properties accompany a progressive loss of biochemical activity in tubulin recruitment and catalyzing MT assembly. Remarkably, phase separation and physical aging in this system are decoupled by a naturally occurring small molecule metabolite abundantly present in the intracellular milieu, L-arginine (L-Arg). Our experiments show that the partitioning of L-Arg into the SynTag-Tau condensates prevents age-dependent changes in the condensate microenvironment, leading to an inhibition of fibril formation. Instead, it facilitates intra-condensate percolation transitions that enhance condensate metastability. L-Arg treatment of SynTag-Tau condensate preserves their activity in microtubule assembly. Finally, we show that L-Arg displays a similar activity to counteract the amyloid formation of untagged WT Tau condensates. Overall, this proof-of-principle study demonstrates that small molecule metabolites can stabilize protein condensates, preventing their transition from a liquid state to amyloid formation and thereby maintaining their function.

## Results and Discussion

### Physical aging of SynTag-Tau condensates leads to the formation of ordered amyloid fibrils

Wild-type (WT) Tau form condensates in physiologically relevant buffer conditions in vitro. These condensates are biochemically active^46^, but they do not transition to amyloid fibrils under quiescent conditions, thereby limiting our investigations (**Supplementary Fig. 1a, b**). Recent reports employing a protein sequence engineering strategy have enabled probing the interplay between phase separation and amyloid formation in an experimentally accessible timescale and resolution that is otherwise difficult to probe^59, 60^. Drawing inspiration from these approaches, we set out to identify a sequence-engineered version of Tau that lowers the nucleation barrier for amyloid formation without substantially affecting its phase separation and function in MT assembly. Previous reports indicate that the fibrillar self-assembly of amyloidogenic proteins can be reinforced by fusing to a compact prionogenic low-complexity sequence^61, 62, 63^. Fusion to prion-like sequences flanking an amyloid-forming protein can act as soluble entropic bristles that favor the prion-like spread of protein aggregates without directly contributing to the primary nucleation mediated by the amyloid core^64^. In the current study, we adapted the strategy of adding a prion-like low-complexity sequence as an N-terminal tag to Tau protein (Fig. 1b). We synthesized SynTag-Tau, which consists of the full-length Tau protein (2N4R isoform) with an amino-terminal tag containing a synthetic prionogenic sequence of 40 amino acid residues^65, 66^ (Fig. 1b; **Supplementary Fig. 2; Supplementary Table 1**). The design of this specific tag is serendipitous. Alphafold3^67^ predicted structure of SynTag-Tau indicates that this prion-like tag and most of the protein are intrinsically disordered, similar to that of the untagged Tau protein (Fig. 1c; **Supplementary Fig. 3**).

We first set out to characterize the formation and maturation of SynTag-Tau condensates utilizing a previously reported protocol of in vitro reconstitution in 10 mM HEPES (pH 7.4), 50 mM NaCl, 0.1 mM EDTA, 2 mM DTT, and a molecular crowder (10% PEG8000)^49, 52^. We tracked age-dependent morphological changes of SynTag-Tau condensates, doped with Atto488 (A488)-conjugated SynTag-Tau, by laser scanning confocal fluorescence microscopy (Fig. 1d). Within 2 to 3 hours after sample preparation, we observed fibrils from SynTag-Tau condensates growing in a heterogeneous manner—some condensates contained visible fibrils, while others did not at this early time point. The fibrils nucleated from one condensate grow into the dilute phase and attach to neighboring condensates, inducing further nucleation and propagation of fibrillar assemblies (Fig. 1d; **Supplementary Video 1**). This observation appears to be consistent with a recent fibril growth model in the presence of condensates^24, 68^ where the growth of fibrils occurs in the dilute phase. Such a process engenders an efflux-generated gradient of protein molecules from nearby condensates to create a network of fibrils with condensates serving as nodes. Shuffling the primary sequence of the prionogenic tag without altering the overall sequence composition in a way that lowered the PLAAC score abrogates condensate aging to fibrils (**Supplementary Fig. 4**). Truncation of the tag sequence to limit it to the portion with the highest weighting in PLAAC score also renders the protein unable to trigger condensate-to-fibril transition (**Supplementary Fig. 5**). Thus, the specific composition and patterning of this synthetic prion-like tag sequence appear to be critical for lowering the nucleation barrier for the condensate-to-fibril transition (Fig. 1e).

We next employed in situ second harmonic generation (SHG), a non-linear label-free optical phenomenon ideal for probing non-centrosymmetric structures with molecular ordering^69^. We observed that SynTag-Tau fibrils, but not SynTag-Tau condensates, contain spatially ordered structures (Fig. 1f). This bears a resemblance to SHG signal originating from bona fide amyloid fibrils observed in the brain of a transgenic model of Alzheimer’s^70^. To evaluate whether these ordered fibrils nucleated by condensates are indeed enriched in cross β-sheet structures, we employed an amyloid-sensitive dye Thioflavin T (ThT). SynTag-Tau fibrils but not nascent SynTag-Tau condensates showed detectable ThT fluorescence (Fig. 1g). To probe the condensate-to-fibril transition in further detail, we measured the (a) timescale for droplet relaxation using a dual-trap optical tweezer, and (b) molecular mobility using fluorescence recovery after photobleaching (FRAP). We observed that the SynTag-Tau condensates undergo an age-dependent dynamical arrest, hallmarked by progressively slower condensate fusion and slower FRAP recovery, prior to the formation of visible amyloid fibrils in the mesoscale (Fig. 1h, i). At the molecular level, we hypothesized a disorder-to-order conformational change of SynTag-Tau is likely to aid in dynamical arrest and concomitant fibrillization. To obtain an age-dependent structural fingerprint of SynTag-Tau molecules in the dense phase, we employed in situ spatially resolved broadband coherent anti-Stokes Raman scattering (BCARS)^71^, which is a label-free hyperspectral Raman imaging technique. Intra-condensate BCARS measurements revealed a progressive increase in the β-sheet content with condensate age, ultimately leading to amyloid fibrils that are predominantly enriched in β-sheet conformers (Fig. 1j; **Supplementary Table 2**).

So far, we have established that SynTag-Tau condensates physically age from a fluid-like phase to fibrillar solids enriched in β-sheet structures. Next, we asked whether these two processes are correlated, meaning how perturbation of SynTag-Tau condensate formation impacts SynTag-Tau fibrillization. Previous literature suggests that electrostatic interactions play a dominant role in Tau phase separation^56, 72, 73^, which can be modulated by tuning the ionic strength of the buffer. We observed that increasing ionic strength diminishes SynTag-Tau phase separation (visualized by globally reduced condensate size). Interestingly, the kinetics of SynTag-Tau fibrillization decrease in a correlated manner (**Supplementary Fig. 6a**). Further, we tested this interplay by titrating the molecular crowder concentration, wherein high [PEG8000] favors protein phase separation propensity via depletion forces^74^. Upon increasing the concentration of the crowder, we find that SynTag-Tau phase separation is enhanced, judged by an increase in condensate volume fraction, and concomitantly, the timescale for condensate-to-fibril transition is reduced (**Supplementary Fig. 6b**). Taken together, these observations suggest that promoting SynTag-Tau condensation enhances fibrillization. Although the insights gained so far seemingly indicate an interplay between phase separation and fibril formation, a key question is how condensate physical aging impacts the function of SynTag-Tau condensates in tubulin recruitment and microtubule (MT) assembly.

### Condensate physical aging impairs biochemical activity

**Figure 2.**
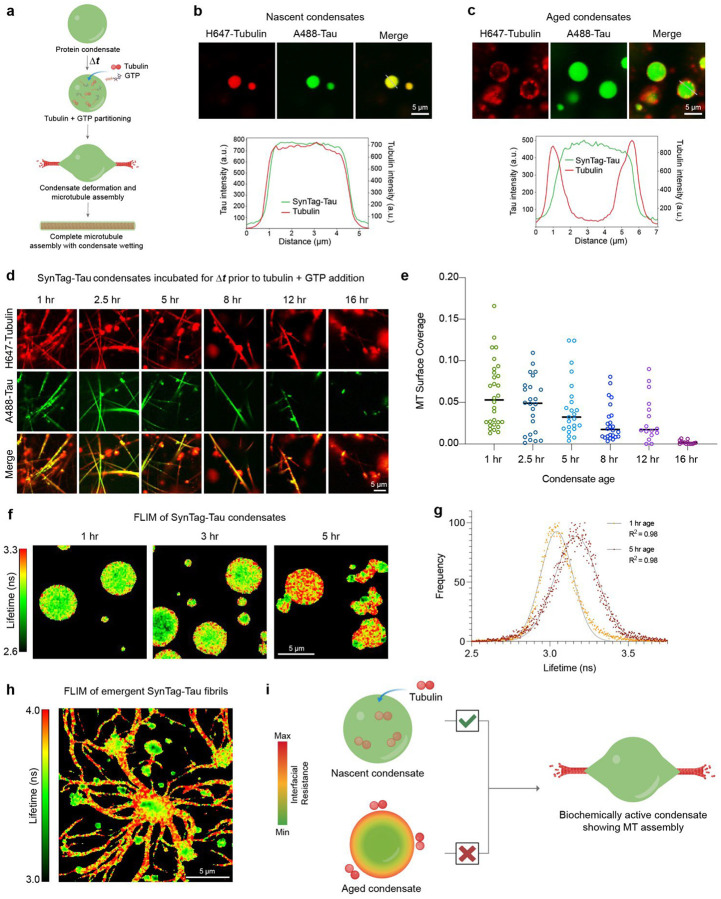
Age-dependent liquid-to-amyloid transition impairs SynTag-Tau condensate activity in microtubule assembly. **(a)** Schematic of tubulin recruitment and microtubule (MT) polymerization in Tau condensates. **(b)** Nascent SynTag-Tau condensates (age = 2 hours) in the presence of 50 nM HiLyte647-labeled tubulin show enrichment of tubulin in the dense phase. Corresponding line profiles are shown below. **(c)** Aged SynTag-Tau condensates (age = 4 hours) with the addition of 50 nM HiLyte647-labeled tubulin show altered tubulin partitioning to the condensate interface. Corresponding line profiles are shown below. **(d)** Condensate age-dependent microtubule polymerization assay in SynTag-Tau condensates. **(e)** Microtubule surface coverage plot corresponding to panel (d). Horizontal lines at each time point represent the median value. The individual data points from replicate experiments are shown. **(f)** Frequency-domain (FD) FLIM images of SynTag-Tau condensates at various timepoints highlight the age-dependent increase in the lifetime of Atto488-labeled SynTag-Tau molecules at the condensate interface, which eventually spreads to the condensate core. **(g)** Representative fluorescence lifetime distributions at 1 hr and 5 hr time points (since sample preparation). The points are the data, and the lines are Gaussian fittings with the corresponding goodness of fit (R^2^) indicated. **(h)** FD-FLIM map of aged SynTag-Tau condensates with emergent fibrillar assemblies (sample age = 8 hours). **(i)** Schematic of condensate physical aging induced interfacial resistance in SynTag-Tau condensates, which perturbs partitioning of tubulin to the condensate dense phase and impairs MT assembly. The concentration of SynTag-Tau protein used in (b, c) is 24 μM with the following buffer composition, 10 mM HEPES (pH 7.4), 50 mM NaCl, 0.1 mM EDTA, and 2 mM DTT along with 10% PEG8000. In (f-h), the concentration of SynTag-Tau protein is 12 μM with the same buffer composition as (b, c). In (d), the concentration of SynTag-Tau used is 12 μM with the following buffer composition, 80 mM PIPES (pH 6.9), 2 mM MgCl_2_, 0.5 mM EGTA, and 2 mM DTT along with 5% PEG8000 crowder. In (d), the tubulin concentration is 5 μM, and the concentration of GTP is 1 mM. Wherever applicable, the concentrations of Atto488-labeled SynTag-Tau and HiLyte647-labeled tubulin are 250 nM and 500 nM (unless specified otherwise), respectively. Each of these experiments was independently repeated at least three times.

We utilized an in vitro tubulin recruitment and MT assembly assay^46^ (Fig. 2a) to quantify the impact of SynTag-Tau condensate aging on biochemical activity. We first tested the partitioning of tubulin alone in the absence of GTP in nascent and aged SynTag-Tau condensates. We find that the nascent condensates can recruit tubulin (labeled with HiLyte647) with homogeneous distribution throughout dense phase (Fig. 2b; **Supplementary Fig. 7**). Contrastingly, tubulin partitioning was attenuated in aged SynTag-Tau condensates with tubulin molecules mostly concentrating close to the interface (Fig. 2c; **Supplementary Fig. 7**). Next, we tested the age-dependent activity of SynTag-Tau condensates using in vitro MT polymerization assay (experimental schematic in Fig. 2a). With increasing condensate age (time = Δ*t*), we observed a gradual reduction in MT polymerization capacity (Fig. 2d, e; **Supplementary Fig. 8, 9, 10**). By the 16-hour time point, the ability of SynTag-Tau condensates to drive MT polymerization ceases completely. The loss of activity of SynTag-Tau condensates with age demonstrates that the condensate material properties affect tubulin recruitment and MT assembly. Similar attenuation of SynTag-Tau condensate activity was observed (**Supplementary Fig. 11**) when we increased the molecular crowder concentration from 5% to 7.5% or 10%, which accelerates SynTag-Tau condensate-to-fibril transition (**Supplementary Fig. 6b**) and was previously shown to modulate condensate material properties^74^.

Recent reports suggest that the interface of protein condensates plays a critical role in nucleating liquid-to-amyloid transition^17, 75, 76^. This is presumed to stem from the unique conformational ensemble of the protein molecules at the condensate interface in contrast to the dilute or dense phase^77^. Therefore, we asked whether SynTag-Tau condensate interfaces play a similar role in fibril formation. To address this, we employed frequency-domain fluorescence lifetime imaging (FLIM) with Atto488-labeled SynTag-Tau. FLIM is a highly sensitive imaging technique that reports on the spatial heterogeneity in the molecular microenvironment of a fluorescent probe. In our experiment, we measured the lifetime of the Atto488-labeled SynTag-Tau to interrogate the local environment of the protein within the condensate as they undergo age-dependent morphological transitions. We find that there is a clear lifetime signature of SynTag-Tau molecules on the interface of the condensates at an early age, which strikingly spreads to the condensate core as the age-dependent transformation of liquid protein condensates to amyloid fibrils takes place (Fig. 2f, g). The lifetimes of SynTag-Tau molecules at the condensate interface are longer than that of the bulk, closer to the lifetimes of the fibrils (Fig. 2h). These data suggest that SynTag-Tau condensate interfaces nucleate amyloid formation. We infer that the altered tubulin partitioning in nascent versus aged protein condensates (Fig. 2b, c) is due to the physical aging of SynTag-Tau condensates that begin at the interface (Fig. 2i). Disabling and/or delaying the fluid-to-fibril transition of protein condensates could potentially protect against condensate loss-of-activity.

### Naturally occurring small molecule metabolites modulate Tau condensate physical aging

**Figure 3.**
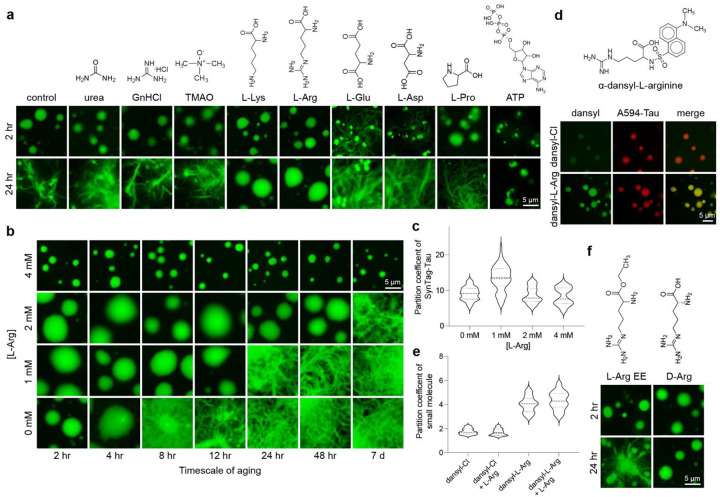
Small molecule metabolites can decouple phase separation and fibril formation in a chemistry-specific manner. **(a)** Effect of naturally occurring small molecule metabolites, chaotropic compounds, and small molecule modulators of protein-protein interactions on phase separation and fibrillization of SynTag-Tau. **(b)** Dose-dependent effect of L-Arg in inhibition of SynTag-Tau condensate-to-amyloid transition. **(c)** The corresponding partition coefficient analysis from fluorescence images as shown in (b). The thick dashed line represents the median, whereas the thinner dotted lines above and below represent the upper and lower quartiles, respectively. **(d)** (top) Chemical structure of α-dansyl-L-arginine (dansyl-L-Arg), a fluorescently labeled analog of L-Arg. (bottom) Partitioning of α-dansyl chloride (dansyl-Cl) or dansyl-L-Arg doped along with L-Arg into SynTag-Tau condensates, visualized using Alexa594- (A594) labeled SynTag-Tau, at 2 hours sample age. **(e)** Partition coefficient analysis of dansyl-Cl or dansyl-L-Arg with/without L-Arg to SynTag-Tau condensates. The thick dashed line represents the median, whereas the thinner dotted lines above and below represent the upper and lower quartiles, respectively. **(f)** L-Arg ethyl ester (L-Arg EE), a derivative of L-Arg that lacks a carboxyl group, failed to prevent condensate aging to fibrils. D-arginine (D-Arg) treated condensates do not transition to fibrils, similar to the L-Arg condition shown in (a). The composition of these samples is 12 μM SynTag-Tau in buffer containing 10 mM HEPES (pH 7.4), 50 mM NaCl, 0.1 mM EDTA, and 2 mM DTT along with 7.5% PEG8000. Small molecules were introduced prior to the induction of protein phase separation, and their concentrations used in these experiments are 2 mM unless specified otherwise. Wherever applicable, the concentration of Atto488-/Alexa594-labeled SynTag-Tau is 250 nM, and the concentration of dansyl-L-Arg/dansyl-Cl is 500 nM (doped with/without L-Arg to make up a total small molecule concentration of 2 mM). Each of these experiments was independently repeated three times.

Identifying small molecule inhibitors of protein fibrillization has been an active area of research for over two decades. In the context of phase separation, small molecules, such as 4,4’-dianilino-1,1’-binaphthyl-5,5’-disulfonic acid (bis-ANS) and adenosine triphosphate (ATP), were shown recently to modulate the phase behavior of ND-linked proteins^26, 32^. These studies and mechanistic perspectives gleaned by quantitative frameworks such as polyphasic linkage^78^ and heterotypic buffering^79, 80^ have postulated that tuning condensate material properties may prevent their aberrant behavior. Motivated by this perspective, we set out to test whether SynTag-Tau condensate maturation timescale can be tuned independently of SynTag-Tau phase separation through small molecule treatment. We first tested generic chemical disruptors of protein-protein interactions and naturally occurring small molecule metabolites at low millimolar concentrations. Remarkably, we found that physiologically abundant cationic amino acids, L-arginine (L-Arg) and L-lysine (L-Lys) inhibit SynTag-Tau condensate-to-fibril formation without perturbing SynTag-Tau phase separation (Fig. 3a). The concentrations at which L-Arg showed such activity is similar to its physiologically relevant concentrations^81, 82^. In the presence of anionic amino acids such as L-glutamic acid (L-Glu) and L-aspartic acid (L-Asp), however, the appearance of SynTag-Tau fibrils seemed to accelerate. The observed tunability of SynTag-Tau liquid-to-fibril transition by these small molecule metabolites is likely owed to their charge and the side chain chemistry as L-proline (L-Pro), which cannot participate in electrostatic interactions, failed to show any modulatory effect (Fig. 3a). The inhibitory effect of L-Arg and L-Lys was also not recapitulated by small molecules with pleiotropic effects on protein-protein interactions, such as those that disrupt protein backbone hydrogen bonding interactions, including guanidinium hydrochloride (GnHCl) and urea (Fig. 3a). The chemical chaperone trimethylamine N-oxide (TMAO) previously reported to disfavor fibrillization of TDP-43^27^ was unable to prevent SynTag-Tau phase separation coupled fibrillization (Fig. 3a). Conversely, ATP appears to hinder fibril formation, however, the condensate size and morphology are substantially affected (Fig. 3a).

We next turned our attention to L-Arg to determine its dose-dependent efficacy in perturbing SynTag-Tau fibrillization (Fig. 3b). At the lowest L-Arg concentration tested (1 mM), the partition coefficient of Atto488-labeled SynTag-Tau molecules in the dense phase is higher compared to the untreated condition. However, increasing the L-Arg concentration to 2 mM or 4 mM did not dramatically alter the partition coefficient relative to the untreated condition (Fig. 3b, c). This data suggests that SynTag-Tau phase separation is not negatively impacted by 1 mM to 4 mM L-Arg. Notably, at 2 mM L-Arg concentration, SynTag-Tau condensate size and morphology looked similar to those of the untreated condition, but the timescale of fluid-to-fibril transition shifted from 8 hours to 7 days (Fig. 3b, c). At 4 mM L-Arg, however, we observed some differences in SynTag-Tau condensate size and surface wetting behavior compared to the untreated conditions (Fig. 3b, c) despite the SynTag-Tau partition coefficient being largely similar. The reduced condensate sizes could be attributed to altered condensate coarsening dynamics. The selective inhibition of fibrillization without abrogating condensate formation reveals a distinction in the molecular driving forces underlying these two processes, indicating that phase separation and fibrillization are biochemically separable. This decoupling phenomenon is distinct from global perturbation to condensate formation, as seen with the effect of modulating ionic strength or molecular crowding (**Supplementary Fig. 6**).

Next, we probed partitioning behavior of L-Arg into SynTag-Tau condensates utilizing α-dansyl-L-arginine (dansyl-L-Arg), a fluorescent analog of L-Arg (Fig. 3d). Using dual-color fluorescence microscopy with SynTag-Tau condensates visualized with Alexa594-conjugated protein, we find positive partitioning with a partition coefficient, k ~ 4 of dansyl-L-Arg within the SynTag-Tau condensate (Fig. 3e). This partition coefficient value is consistent with the known partition coefficients of similar small molecule metabolites in condensates^39^. The partitioning of dansyl-L-Arg is not due to the dansyl group as partitioning of α-dansyl chloride to SynTag-Tau condensates is much weaker (k ~ 1.5) (Fig. 3d, e).

To investigate how the inhibitory effect of L-Arg on SynTag-Tau condensate aging is related to its chemical properties, we tested L-Arg ethyl ester (L-Arg EE) and observed no impact on SynTag-Tau condensate aging (Fig. 3f). Experiments with D-arginine (D-Arg) yielded similar results as L-Arg, revealing that the stereospecificity of arginine is not critical to its inhibitory activity (Fig. 3f). Next, we tested whether multivalent polymers of lysine or arginine as well as charged polyamines such as spermine and spermidine could impose an enhanced inhibitory potential to condensate-to-fibril transformation relative to small molecules (L-Arg/L-Lys). Intriguingly, treating condensates with multivalent cationic molecules did not offer any improvement in fibrillization timescale relative to the small molecule treatment (**Supplementary Fig. 12;** Fig. 3a). Therefore, we conclude that the observed inhibitory activity of L-Arg, close to its physiological concentration, is chemistry-, valence- and charge-specific, but does not depend on the chirality.

### L-Arg prevents cross β-sheet formation and nucleation of fibrils at the condensate interface

**Figure 4.**
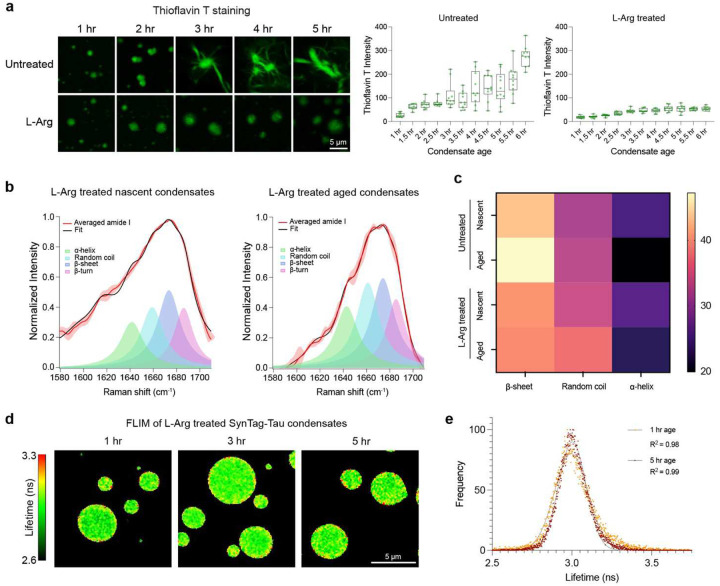
L-Arg prevents cross β-sheet formation and nucleation of fibrils at the condensate interface. **(a)** ThT fluorescence of condensates at different sample ages without (untreated) and with 2 mM L-Arg. Corresponding plots of ThT fluorescence intensities are shown on the right. The center line represents the median and the individual data points from replicate experiments are shown. **(b)** Peak fitting of averaged amide I spectra obtained from BCARS hyperspectral imaging of L-Arg-treated SynTag-Tau condensates reveals changes in protein molecular conformations from 2 hours of age (nascent condensates) to 6 hours of age (aged condensates). **(c)** Heat map based on the amide I spectra obtained through BCARS hyperspectral imaging showing the mean percentages of protein conformations in untreated versus 2 mM L-Arg treated SynTag-Tau condensates at 2 hours (nascent) and 6 hours (aged) of age. The mean percentages represent the quotient of each conformation’s peak fitted area divided by the cumulative area of β-sheet, random coil, and α-helix. **(d)** FD-FLIM map of L-Arg treated condensates at various time points (since sample preparation). **(e)** Representative fluorescence lifetime distributions at 1 hr and 5 hr time points (since sample preparation). The points are the data, and the lines are Gaussian fittings with the corresponding goodness of fit (R^2^) indicated. The composition of these samples is 12 μM SynTag-Tau in buffer containing 10 mM HEPES (pH 7.4), 50 mM NaCl, 0.1 mM EDTA, and 2 mM DTT along with 10% PEG8000 crowder. The L-Arg concentration used in these experiments is 2 mM. Wherever applicable, the concentration of Atto488 labeled SynTag-Tau is 250 nM, and the concentration of ThT is 50 μM. Each of these experiments was independently repeated three times.

Given that L-Arg can prevent age-dependent changes in SynTag-Tau condensate morphology (Fig. 3), we next sought to probe its effect on the structure and dynamics of SynTag-Tau condensates. Using ThT fluorescence assay as a function of time, we observed that the age-dependent increase in the signal originating from amyloid fibrils is notably less pronounced for condensates in the presence of L-Arg (Fig. 4a). This observation suggests that L-Arg inhibits conformational conversion of disordered SynTag-Tau molecules within condensates to cross-β-rich conformers, preventing nucleation of amyloid fibrils. For further examination, we employed BCARS hyperspectral imaging and observed that the amide I band of SynTag-Tau in nascent condensates is fairly similar in the absence and presence of L-Arg (Fig. 4b, c; **Supplementary Table 2**). However, the amide I band of aged SynTag-Tau condensates treated with 2 mM L-Arg appears distinct from that of untreated condensates at the same age. The most dramatic difference is the decrement in the mean population of β-sheet conformers in L-Arg-treated condensates (39.3%) versus the untreated condition (47.2%). We also note a moderate increase in non-β conformations in small molecule-treated condensates relative to untreated condensates, namely random coil (37.4% vs. 32.9%, respectively) and α-helix (23.3% vs. 19.9%, respectively) (Fig. 4c). This may suggest an exciting possibility that L-Arg might slow down the SynTag-Tau condensate liquid-to-fibril transition by populating alternative ensemble conformational states of SynTag-Tau. Follow-up work is necessary to explore this possibility further.

To probe the spatially resolved SynTag-Tau condensate microenvironment with age in the presence of L-Arg, we performed FLIM measurements using an identical approach as outlined in Fig. 2f. When comparing with the SynTag-Tau condensates without L-Arg, we made two key observations. The first is that the lifetime of fluorescently labeled SynTag-Tau molecules does not change over the same timescale, and the interface of these condensates appears similar in terms of the fluorescence lifetime with respect to the SynTag-Tau molecules at the condensate core (Fig. 4d, e). These data collectively suggest that the mesoscale changes in the SynTag-Tau condensate morphology with age are inhibited by L-Arg. Finally, we tested the efficacy of L-Arg in the heparin-induced maturation of WT Tau condensates into amyloid fibrils. L-Arg proved effective in this in vitro model of condensate aging as well, indicating that the selective inhibitory potential of L-Arg against condensate-to-fibril transition does not stem from interactions between L-Arg and the synthetic prionogenic tag in SynTag-Tau but potentially frustrating the interactions mediated by the zipper-motifs in Tau (**Supplementary Fig. 13**).

### Small molecule-induced enhancement in condensate viscoelasticity weakens fibrillization propensity

**Figure 5.**
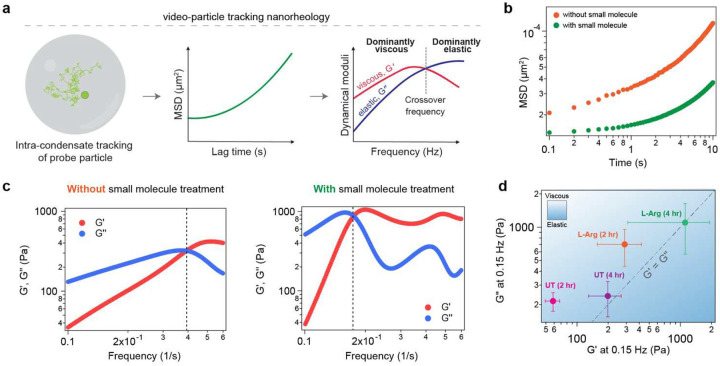
Small molecule-mediated enhancement of condensate viscoelasticity. **(a)** Schematic of VPT nanorheology measurements using 200 nm probe particles passively embedded within Tau condensates. **(b)** Representative mean squared displacement (MSD) measurements of probe particles inside Tau condensates with or without small molecule treatment (2 mM L-Arg). The measurements were conducted 2 hours after sample preparation. **(c)** Estimated dynamical moduli of Tau condensates in the absence (left) or presence (right) of 2 mM L-Arg. The measurements were conducted 2 hours after sample preparation. The dashed line represents the crossover frequency. **(d)** Reports a diagram-of-states for untreated (UT) and small molecule treated (L-Arg) Tau condensates at the indicated sample age, based on the measured moduli at 0.15 Hz. The experimentally determined data are represented as mean values with standard deviations. The composition of the samples used here is 40 μM Tau in buffer containing 10 mM HEPES (pH 7.4), 50 mM NaCl, 0.1 mM EDTA, and 2 mM DTT along with 7.5% PEG8000, and 6.25 μM heparin. Wherever applicable, the L-Arg concentration used in experiments is 2 mM. Each of these experiments was independently repeated three times.

The effect of L-Arg in regulating condensate-to-fibril transition may stem from modulating the material properties of Tau condensates. To test this idea, we employed video particle tracking (VPT) nanorheology using 200 nm-sized fluorescent probe particles passively embedded inside WT Tau condensates (Fig. 5a). From the mean squared displacements (MSDs) of the fluorescent probes, we computed the frequency-dependent viscoelastic moduli of Tau condensates. The timescale of the dominantly viscous versus dominantly elastic behavior of Tau condensates can be deduced by estimating the crossover frequency from the frequency-dependent viscoelastic moduli. In the absence of L-Arg, Tau condensates are highly viscoelastic, with probe particles exhibiting sub-diffusive motion (diffusivity coefficient, α<1) within the experimental timescale (Fig. 5b, c). With aging, these condensates exhibit a progressive lowering of the crossover frequency and increase in the storage (G’) and loss (G”) moduli, suggesting an increase in the network elasticity (Fig. 5d; **Supplementary Fig. 14**). Surprisingly, L-Arg-treated condensates at 2 hours of age were found to be more viscoelastic relative to that of the untreated condition (Fig. 5b, c). At 4 hours, these condensates exhibit a substantial increase in viscoelasticity (Fig. 5d; **Supplementary Fig. 14**). These data suggest a surprising role of L-Arg in strengthening intra-condensate networking interactions, otherwise referred to as percolation^83, 84^. Optical tweezer-based active fusion measurements of SynTag-Tau condensate show a consensus with nanorheology measurements (Fig. 5b **vs. Supplementary Fig. 15a, Fig. 1h**) in that L-Arg leads to a slowdown in condensate fusion speed in a dose-dependent manner. In agreement with these results, FRAP measurements at longer timescales revealed progressively slower recovery traces for L-Arg-treated condensates (**Supplementary Fig. 15b**). In total, measurements of condensate material properties reveal that L-Arg suppresses condensate-to-fibril transition via enhancement of condensate viscoelasticity.

### Selective inhibition of condensate-to-fibril transition preserves condensate biochemical activity

**Fig. 6.**
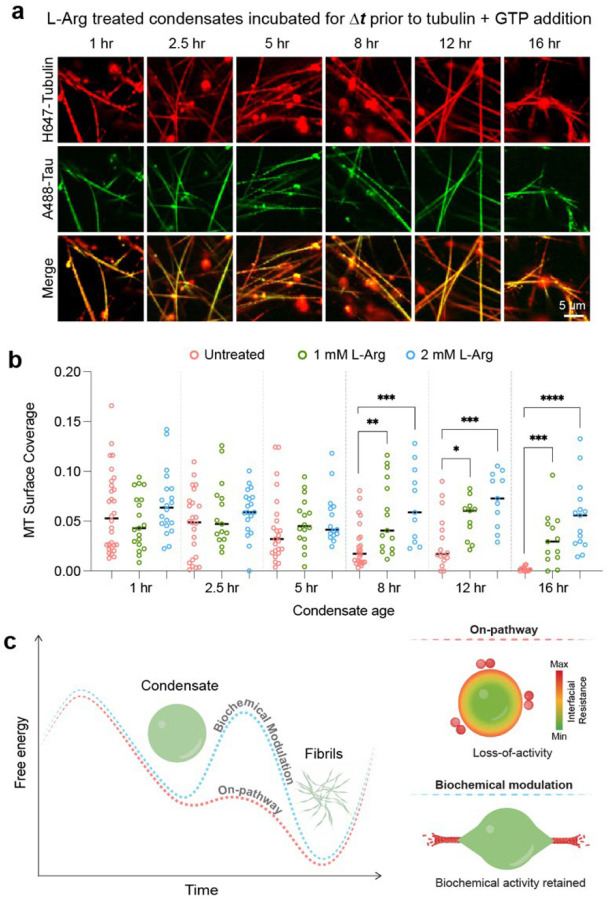
Selective inhibition of condensate age-dependent fibril formation preserves condensate biochemical activity. **(a)** Microtubule polymerization assay of SynTag-Tau condensates treated with 1 mM L-Arg at various time points. **(b)** A comparison of MT surface coverage as a function of condensate age between untreated SynTag-Tau condensates (Fig. 2e) and L-Arg treated SynTag-Tau condensates (images shown in **Supplementary Fig. 16**). Horizontal black lines represent the median value. The individual data points from replicate experiments are shown. Statistical significance was determined using an unpaired two-sided Student’s *t*-test between the MT surface coverages of untreated condensates (orange) and either 1 mM L-Arg treated condensates (green) or 2 mM L-Arg treated condensates (blue) (* means p<0.05, ** means p<0.01, *** means p<0.001, **** means p<0.0001). The associated *P* values are shown from left to right: 0.0028, 0.0003, 0.0264, 0.0006, 0.0002, and 0.00009. **(c)** Schematic representing the proposed model for the biochemical modulation of energy barrier governing protein condensate-to-fibril transition and their impact on the condensate biochemical activity. The composition of samples used in panels (a) and (b) is 12 μM SynTag-Tau protein in buffer containing 80 mM PIPES (pH 6.9), 2 mM MgCl_2_, 0.5 mM EGTA, and 2 mM DTT along with 5% PEG8000 crowder. The tubulin concentration used here is 5 μM, and the concentration of GTP is 1 mM. The concentrations of Atto488-labeled SynTag-Tau and HiLyte647-labeled tubulin are 250 nM and 500 nM, respectively. These experiments were independently repeated three times.

The effect of L-Arg showing protective effects against condensate-to-fibril transition of SynTag-Tau motivated us to test its role in MT assembly (Fig. 2). We observe that similar to untreated SynTag-Tau condensates, the L-Arg-treated condensates can assemble MTs with comparable efficiency up to 5 hours of physical aging (Fig. 6a, b; **Supplementary Fig. 16**). After the 5-hour mark, these trends start to diverge. The untreated SynTag-Tau condensates progressively lose their MT assembly activity as seen by the reduced MT formation (Fig. 2d, e). In contrast, the L-Arg-treated SynTag-Tau condensates (1 mM and 2 mM L-Arg) show a steady trend in MT assembly activity in the same timescale without appreciable decline. Particularly, at the 16-hour time point, the untreated SynTag-Tau condensates completely lose activity, whereas the L-Arg treated condensates continue to retain their function (Fig. 6a, b; **Supplementary Fig. 16**). With our overall observations, we posit that nucleation of amyloid assemblies at the SynTag-Tau condensate interface results in the accumulation of tubulin at the condensate interface, hindering MT assembly. In the presence of L-Arg, fibril nucleation at the condensate interface is inhibited, allowing favorable partitioning of tubulin to the condensate core and facilitating tubulin polymerization (Fig. 6c).

## Conclusion

Previous reports have suggested that liquid-like biomolecular condensates may act as hubs for the aggregation of disease-linked proteins^20, 22, 85, 86^. An important question in this regard is whether the driving forces for phase separation and amyloid fibril formation are the same or merely overlapping. If condensates were to be crucibles for amyloid fibril formation^87^, one would speculate that physicochemical factors that stabilize the condensed phase of proteins would result in a commensurate acceleration in fibril formation. Using sequence engineering of the prion-like domain of hnRNPA1, recent reports have independently shown the separation of functions−the separability of sequence features that drive phase separation and fibril formation^17, 24^. In our studies, we demonstrate an orthogonal approach to the separation of function paradigm by utilizing small molecules to modulate the material properties of protein condensates. Although protein sequence engineering has been a common way to dissect the interplay between condensate formation and aging into stable solids, such approaches are not feasible to apply in disease models. Our results provide a proof-of-principle that the energy barrier for condensate-to-fibril transition can be selectively modulated by small molecules (Fig. 6c), thereby preserving condensate biochemical activity.

What is the mechanism of L-Arg’s action in inhibiting Tau condensate-to-fibril transition? Firstly, we find that the Tau condensate interface plays a critical role in nucleating fibril formation, consistent with other protein systems that show similar aging behavior^17, 75, 76^. Therefore, the mechanism of L-Arg’s action may rest, in part, on its ability to prevent amyloid nucleation at the Tau condensate interface, a process that leads to a crust-like solid shell formation in aged condensates^88^. Inhibition of the solidification of the interface of Tau condensates would enable favorable partitioning of tubulin to condensate core, allowing MT assembly. Secondly, L-Arg strengthens the metastable viscoelastic network of Tau condensates, thereby delaying the onset of the condensate-to-fibril transition. A similar mechanism has recently been proposed in the instance of Tau condensates treated with methylene blue^57^, a long-studied drug candidate in clinical trials of Tauopathies^89^. Within the intracellular environment, L-Arg concentration is in the lower millimolar range^81, 82^, similar to that used in our study, which leads us to speculate that L-Arg may act as a natural regulator of aberrant protein phase transitions, similar to the proposed role of ATP as a biological hydrotrope^26^. In contrast, we found that some other small molecule metabolites, such as L-Asp and L-Glu, enhance the Tau liquid-to-solid transition. These findings inspire a hypothesis that the cellular metabolic state itself is a critical regulator of protein phase separation and aggregation in living systems. Under diverse conditions, cells can produce a variety of metabolites that can dynamically tune the aging dynamics of biomolecular condensates.

The current study employs a unique strategy of introducing a synthetic prionogenic sequence tag to full-length Tau protein, which lowers the nucleation barrier of condensate-to-fibril transition without perturbing condensate formation and their biochemical activity. Our findings are in corroboration with growing lines of evidence that have directly correlated the impact of disease mutations to the weakening of condensate metastability and, inversely, global stabilization of the fibrillar solid phase^12, 24, 90^. These observations warrant further investigations on the molecular grammars of condensate metastability and aging timescales^91^. The designed Tau protein system used in the study provided valuable insight into the effect of material properties and physical aging on condensate biochemical activity.

In summary, protein condensates are metastable fluids that must overcome a free-energy barrier to transition to fibrils, which is a more thermodynamically stable state (Fig. 6c). Our study reinforces the framework of separation of functions of protein phase separation and physical aging into irreversible solids. We experimentally demonstrated that these two processes can be decoupled by small molecules that selectively heighten the nucleation barrier for condensate-to-amyloid transition. Our results may inspire strategies to selectively target protein pathological fibrillization without perturbing their functional phase separation.

## Data Availability

All data are available in the manuscript or the supplementary materials.
